# 

**Published:** 1872-08

**Authors:** 


### BIBLIOGRAPHICAL.

*Amnesic and Ataxic Aphasia with Agraphia and Temporary Right Hemiplegia.* The Result of Embolism of the Left Middle Cerebral Artery. By T. M. B. Cross, M.D., of Bellevue Hospital, N. Y., Printed by Jno. B. Morton & Co. Louisville, 1872. The history, symptoms, and successful treatment of an interesting case which is attributed to embolism, the paralysis being on the right side of the body, while the lesion was on the left side of the brain.

*The Question of Quarantine.*—The Nature and Prevention of Communicable Zymotic Diseases. A paper read before the Medical Library and Journal Association of New York. By Alfred L. Carroll, M.D. T. Leypoldt, publisher, 712 Broadway, N. Y., 1872.

*The Farm and Fireside Journal.*—Devoted to the culture of the soil, and of the mind; and adapted to the wants of every honsehold. A beautiful work of art as a premium to every subscriber. Terms:—One dollar per annum in advance. Published by "The Farm and Fireside Association," 12 Pine Street New York.

*The American Land and Law Adviser.*—The (Pittsburgh Pa.,) *Real Estate Register* comes to us this week enlarged to a beautiful sixteen page, sixty-four column, illustrated weekly, with the name changed to the *American Land and Law Adviser.* The original features introduced into the old paper by its publishers caused it to be sought after by persons in all parts of the United States, and thus encouraged by public patronage, the publishers determine to give to the people a paper every way worthy of the name they have chosen for their new weekly. The *American Land and Law Adviser* is a "*Weekly Journal of Real Estate, Finance, Building, and Popularization of Law.*" The issue before us is *absolutely a necessity* to every landed proprietor or real estate owner in the country, as well as to every citizen in the United States that wishes to keep posted on that indestructible element of value—*Real Estate.* The law department of this excellent weekly is edited by the ablest law councellors in the country, and answer, *free of charge*, all questions of law submitted to the paper with a clearness and accuracy that makes them understood by men of the most ordinary intelligence. This feature alone should cause it to be taken by every farmer and land owner in the country. The illustrations on the first page of original designs for cottages and suburban residences, gotten up expressly for this journal, is also a feature that commends itself to those about to build, and if we are to judge the future by the first issue, now before us, we should say it alone was worth many times more than the subscription price. The weekly correspondence from the General Land Office at Washington. D. C., giving the latest laws governing the Public Lands, Homestead and Pre-emption, as well as those from all parts of the country,—is also a valuable feature; to say nothing of its news and general information found in no other journal in the United States. To crown all, the enterprising publishers offer, by way of inducing an examination and subscription, a beautiful $5.00 Chromo of either of the following subjects: "*The Lost Babe,*" or "*The Unwelcome Visitor*;" *all* for the exceedingly low price of $2.50 a year, embracing a beautiful parlor picture and over 800 pages of useful reading matter, and illustrations. We would say to all our readers, send stamp for a sample copy. Address Croft & Philips, Publishers *American Land and Law Adviser*, Pittsburgh, Pa.

---

